# Determining the Traditional Chinese Medicine (TCM) Syndrome with the Best Prognosis of HBV-Related HCC and Exploring the Related Mechanism Using Network Pharmacology

**DOI:** 10.1155/2021/9991533

**Published:** 2021-06-29

**Authors:** Zhulin Wu, Chunshan Wei, Lianan Wang, Li He

**Affiliations:** The Fourth Clinical Medical College of Guangzhou University of Chinese Medicine (Shenzhen Traditional Chinese Medicine Hospital), Shenzhen 518033, China

## Abstract

**Background:**

In traditional Chinese medicine (TCM), TCM syndrome is a key guideline, and Chinese materia medicas are widely used to treat hepatitis B virus- (HBV-) related hepatocellular carcinoma (HCC) according to different TCM syndromes. However, the prognostic value of TCM syndromes in HBV-related HCC patients has never been studied.

**Methods:**

A retrospective cohort of HBV-related HCC patients at Shenzhen Traditional Chinese Medicine Hospital from December 2005 to October 2017 was analyzed. The prognostic value of TCM syndromes in HBV-related HCC patients was assessed by Kaplan–Meier survival curves and Cox analysis, and the TCM syndrome with the best prognosis of HBV-related HCC patients was determined. To further study the relevant mechanisms, key Chinese materia medicas (KCMMs) for the TCM syndrome with the best prognosis were summarized, and network pharmacology was also performed.

**Results:**

A total of 207 HBV-related HCC patients were included in this research, and we found that HBV-related HCC patients with TCM excess syndrome had better OS. Then, a total of eight KCMMs for TCM excess syndrome were identified, whose crucial ingredients included quercetin, beta-sitosterol, kaempferol, luteolin, and XH-14, and KCMMs could play a therapeutic role through MAPK, JAK-STAT, Wnt, Hippo, and other pathways. Moreover, TP53, SRC, STAT3, MAPK3, PIK3R1, HRAS, VEGFA, HSP90AA1, EGFR, and JAK2 were determined as the key targets.

**Conclusion:**

We propose a new research method of “prognosis of TCM syndromes-KCMMs-network pharmacology” to reveal the prognostic value of TCM syndromes and the potential mechanism by which TCM syndromes affect prognosis.

## 1. Introduction

Hepatocellular carcinoma (HCC), the major type of liver cancer, is the fifth most prevalent malignant tumor with more than 800,000 new cases and deaths yearly [1]. Hepatitis B virus (HBV) is the principal etiological risk factor for HCC, especially in developing countries [2], and the incidence and mortality of HCC in China account for about 50% of global cases [3]. Moreover, current multitargeted tyrosine kinase inhibitors for HCC show limited efficacy and adverse effects [4], and the survival rate of five years for patients with HCC remains relatively low [5]. Also, due to the high level of malignancy and late diagnosis, HCC patients usually lose the opportunity to undergo radical treatment, and most of them can only receive palliative care [5].

Traditional Chinese medicine (TCM), as complementary and alternative medicine, has become a frequently used anticancer therapy for HCC in China, and it can even be used throughout the whole process of HCC. Recently, it has been found that TCM can inhibit angiogenesis, reduce the microvessel density of HCC, and improve abnormal liver-regenerating microenvironment, thus delaying the development of HCC [6]. Moreover, there were significant differences in prognosis between HCC patients with TCM use and HCC patients without TCM use [7]. However, the prognostic value of TCM syndromes in patients with HBV-related HCC remains unclear, and it is unknown which of TCM syndrome has the best prognosis in HBV-related HCC and how this TCM syndrome influences the prognosis. As an adjuvant therapy, Chinese materia medica is widely used to ameliorate the quality of life and survival time of HBV-related HCC patients in China, and prescriptions of Chinese materia medicas are mainly formulated based on the TCM syndrome types. Therefore, the key Chinese materia medicas (KCMMs) corresponding to TCM syndrome types can be used to explore the potential mechanisms by which TCM syndromes affect prognosis. In this study, based on a new research method of “prognosis of TCM syndromes-KCMMs-network pharmacology,” we identified the TCM syndrome with the best prognosis of HBV-related HCC and then studied the potential molecular mechanisms.

## 2. Materials and Methods

### 2.1. Patients with HBV-Related HCC and Study Design

A retrospective study was carried out on a cohort of patients who were clinically or pathologically diagnosed with HBV-related HCC between December 2005 and October 2017 at Shenzhen Traditional Chinese Medicine Hospital (Shenzhen, China). Patient inclusion criteria should be as follows: patients with HBV-related HCC and the diagnoses of HCC were confirmed pathologically or clinically based on “guidelines for diagnosis and treatment of primary liver cancer in China (2019 edition)” [8]; patients were treated with TCM (Chinese materia medicas); survival status of patients can be obtained; and survival time greater than 4 weeks. Exclusion criteria were listed as the following: patients who had other malignancies; patients without hepatitis B; and patients with incomplete clinical information or survival data. Our study was censored on July 31, 2020, and ethical approval (K2020-090-01) was acquired from the Ethics Committee of Shenzhen Traditional Chinese Medicine Hospital.

### 2.2. Diagnosis of TCM Syndromes and Prognostic Analysis

In TCM theory, TCM syndrome consists of a series of specific clinical symptoms, but there is no unified diagnostic standard of TCM syndromes currently. In our study, TCM syndromes of HBV-related HCC patients were determined on the basis of the “guidelines for diagnosis and treatment of primary liver cancer in China,” [8] the standard of excess and deficiency syndromes in “*Diagnostics of TCM*” (a textbook for universities and colleges of TCM in China), and a previous study [9]. In our study, TCM syndromes were classified into three types: excess syndrome, deficiency syndrome, and syndrome of intermingled deficiency and excess patterns. According to the previous studies, symptoms such as tiredness, weakness, poor appetite, diarrhea, clear urine in large amounts, weak pulse, and pale tongue indicate deficiency syndrome, and dry mouth, bitter taste in mouth, deep-colored urine, sticky and greasy in feces, red tongue, yellow greasy fur, and stringy pulse suggest excess syndrome. Additionally, the syndrome of intermingled deficiency and excess pattern has the symptoms of deficiency syndrome and excess syndrome. Moreover, all relevant data were extracted from the patients' medical records at the first diagnosis, and clinical information collected for analysis was as follows: age, the TCM syndrome type, gender, tumor number and size, Child–Pugh classification, ALBI (albumin-bilirubin) score, HBV-DNA viral load, information on whether the participant underwent surgery, survival data, and TCM prescriptions. Specifically, the ALBI grade of each patient was determined according to the previous research [10]. TCM syndromes obtained from the medical records were confirmed independently by two professional physicians (Lianan Wang and Li He), and any disagreement was resolved by discussion with a third independent physician (Chunshan Wei).

In the present study, overall survival (OS) was utilized to assess prognostic indicators, and OS was defined as the time to death for any reason. Clinical variables were grouped on the basis of clinical experience and previous studies. TCM syndromes and clinical variables were subjected to Cox regression analyses. Besides, Kaplan–Meier (KM) survival analysis was used to evaluate the prognostic value of TCM syndromes.

### 2.3. TCM Prescriptions' Collection and Extraction of Key Chinese Materia Medicas (KCMMs)

TCM prescriptions of Chinese materia medicas are mainly formulated based on the TCM syndrome types, so KCMMs were utilized to study the mechanisms that TCM syndromes influence the prognosis of HBV-related HCC. TCM prescriptions for the treatment of HBV-related HCC patients with excess syndrome were collected. Then, SPSS Modeler 15.0 (SPSS Inc., Chicago, IL, USA) was employed to calculate frequencies and perform association rule analysis, and the KCMMs of the TCM prescriptions were extracted according to the association rule analysis. In our study, KCMMs were identified under the conditions of minimum antecedent support = 10%, minimum rule confidence = 80%, maximum number of antecedents = 2, and number of links ≥ 30.

### 2.4. Screening Bioactive Ingredients and Targets of KCMMs

Traditional Chinese Medicine Systems Pharmacology Database and Analysis Platform (TCMSP, https://tcmspw.com/tcmsp.php) [11] was applied to obtain the bioactive ingredients of KCMMs. The bioactive ingredients were selected under the conditions of drug-likeness ≥0.18 and oral bioavailability ≥30% [12]. The corresponding targets of bioactive ingredients were predicted using TCMSP, SwissTargetPrediction (http://www.swisstargetprediction.ch/) [13], Encyclopaedia of Traditional Chinese Medicine (ETCM, http://www.tcmip.cn/ETCM/index.php/Home/) [14], and SymMap (https://www.symmap.org/) [15]. For SwissTargetPrediction, the predicted targets with a probability value >0.1 were chosen. After that, we convert all targets into corresponding target gene names using the UniProt Knowledgebase (https://www.uniprot.org/).

### 2.5. Acquisition of Disease-Related Targets and Construction of the Network

For a more comprehensive study of mechanisms, potential therapeutic targets related to HCC were extracted using “HBV hepatocellular carcinoma” and “HBV HCC” as the keyword from the GeneCards (https://www.genecards.org/) and OMIM databases (https://omim.org/) [16]. In GeneCards, only target genes with relevance scores >10 were included in the analysis. Disease-related targets were obtained after eliminating duplication. The common target genes of KCMMs and HBV-related HCC were screened using Venn diagrams (http://bioinformatics.psb.ugent.be/webtools/Venn/). Then, the KCMM bioactive ingredients-common target genes network was built by using Cytoscape (https://cytoscape.org/, version 3.7.1).

### 2.6. GO Term Enrichment and KEGG Pathway Analyses of Common Target Genes

In the present study, Gene Ontology (GO) and Kyoto Encyclopaedia of Genes and Genomics (KEGG) signaling pathway analyses for common target genes of KCMMs and HBV-related HCC were carried out by Metascape (http://metascape.org) [17]. In Metascape, the analytical conditions were as follows: min overlap = 3, min enrichment = 1.5, and *P* value = 0.01.

### 2.7. PPI Interaction Analysis and Survival Analysis of Hub Genes

Search Tool for the Retrieval of Interacting Genes (STRING) database (https://string-db.org/) [18] was utilized to establish the protein-protein interaction (PPI) network of the common target genes, the minimum required interaction score was set to 0.900 (highest confidence), and the species was limited to “*Homo sapiens*.” Then, the presentation of the PPI network was performed using Cytoscape 3.7.1. Based on the results of PPI, CytoHubba [19], a plugin in Cytoscape 3.7.1, was used to obtain the top 15 target genes with the highest degree (number of connections). Besides, the prognostic values of hub genes were assessed by Kaplan–Meier plotter (http://www.kmplot.com) [20], which contains survival data and mRNA expressions of 364 HCC patients from the Cancer Genome Atlas (TCGA).

### 2.8. Statistical Analysis

**Figure 1:**
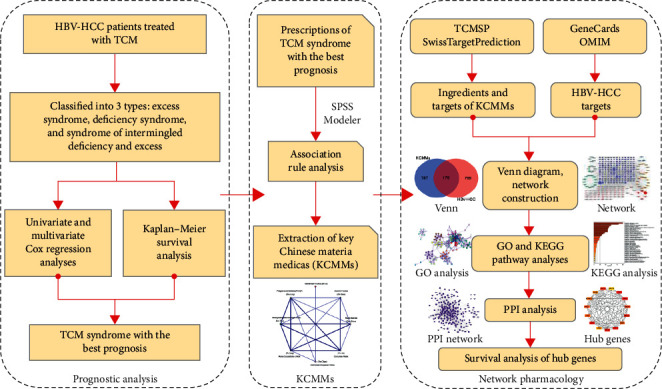
The flowchart of our study design.

Correlations between TCM syndromes and OS were analyzed by KM curves and the log-rank test using “survival” packages of R, version 4.0.3. Categorical variables were given as percentages, nonnormal distribution data were shown as median (interquartile range, IQR), and normally distributed variables were expressed as mean (standard deviation, SD). In Cox regression analyses, the effect of variables was reported as hazard ratio with 95 percent confidence intervals (HR, 95% CI). In our study, significance was accepted when the *p* value was less than 0.05 (*p* < 0.05). The workflow of our research is illustrated in Figure 1.

## 3. Results

### 3.1. Clinical Features and OS of the Included Patients

In total, 207 HBV-related HCC patients who fulfilled the inclusion criteria were included in our study. The clinical characteristics and TCM syndromes of HBV-related HCC patients in the cohort are reported in Table 1. The mean age (SD) of the 207 HBV-related HCC patients was 55.05 (12.22) years old, ranging from 25 to 81 years, and the TCM syndrome types of HBV-related HCC were deficiency syndrome pattern (*n* = 36; 17.39%), excess syndrome pattern (*n* = 98; 47.34%), and syndrome of intermingled deficiency and excess pattern (*n* = 73; 35.27%). Moreover, the median OS time of all HBV-related HCC patients was 730 days (range: 30 to 5133 days).

### 3.2. Results of Survival and Cox Regression Analyses

**Figure 2:**
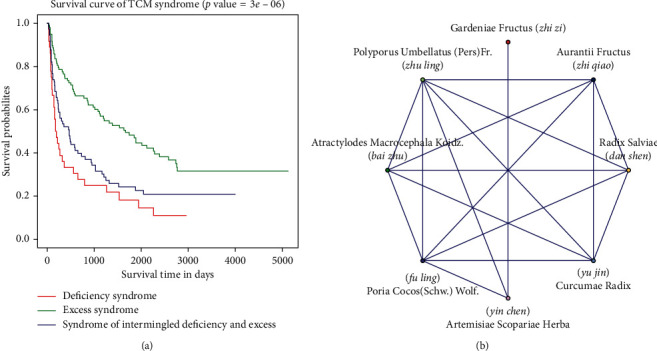
Kaplan–Meier plot of traditional Chinese medicine (TCM) syndromes and the network of key Chinese materia medicas (KCMMs). (a) Survival curves of HBV-related HCC patients with distinct TCM syndromes. (b) The network of eight KCMMs, involving 18 edges. Based on the association rules, each edge represents a combination.

Results of the univariate and multivariate Cox regression analyses are presented in Table 1, and the distribution of TCM syndromes is also displayed in Supplementary Table S1. In the univariate Cox regression analysis, except for gender, all of the other factors were related to OS. Subsequently, clinical variables with *p* value <0.1 were further analyzed in the multivariate Cox regression model, and we found that TCM syndromes (excess syndrome), tumor size, tumor number, no surgery, and Child–Pugh classification were independent risk factors for OS time (Table 1). In TCM syndromes, KM survival curves showed that there was a trend toward better OS in patients with the excess syndrome pattern (*p* value < 0.05), and the result is summarized in Figure 2(a).

### 3.3. KCMMs for HBV-Related HCC with Excess Syndrome

Based on the result of the survival analysis, HBV-related HCC patients with excess syndrome had longer survival. To further study the possible mechanisms, Chinese materia medicas of TCM prescriptions for HBV-related HCC patients with excess syndrome were analyzed. A total of 98 TCM prescriptions in treating HBV-related HCC were obtained, involving 124 Chinese materia medicas (Supplementary Table S2). Then, eight KCMMs were acquired using SPSS Modeler 15.0, including *Poria cocos* (Schw.) Wolf. (Fu Ling), *Atractylodes macrocephala* Koidz. (Bai Zhu), *Polyporus umbellatus* (Pers) Fr. (Zhu Ling), *Radix salviae* (Dan Shen), *Artemisiae scopariae* Herba (Yin Chen), *Aurantii fructus* (Zhi Qiao), *Curcumae radix* (Yu Jin), and *Gardeniae fructus* (Zhi Zi). Furthermore, the top 15 combinations of Chinese materia medicas and the network of the eight KCMMs are displayed in Table 2 and Figure 2(b), respectively.

### 3.4. Bioactive Ingredients and Targets of KCMMs and Disease-Associated Target Genes

**Figure 3:**
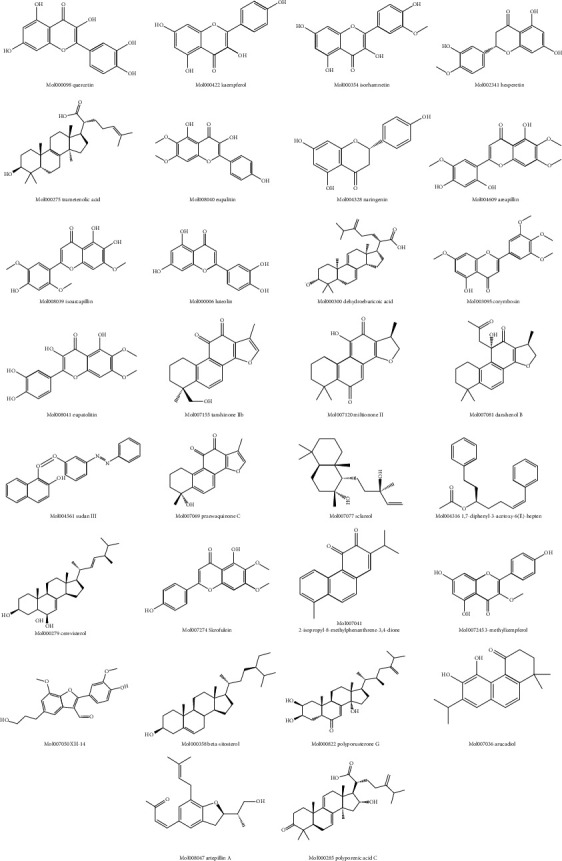
Chemical structures of top 30 ingredients of KCMMs according to the number of targets.

After removing the repeating ingredients, a total of 127 bioactive ingredients of KCMMs were obtained, and 1052 target genes of KCMMs were identified; the detailed data are presented in Supplementary Table S3. Additionally, the information of the top 30 bioactive ingredients of KCMMS is shown in Table 3 according to the number of targets, and chemical structures of these ingredients were drawn through ChemDraw, version 17.1 (PerkinElmer, USA) (Figure 3). As for disease-associated target genes, a total of 978 potential therapeutic target genes for HCC (804 in GeneCards and 195 in OMIM) were acquired after removing duplication.

### 3.5. Network of Bioactive Ingredients-Common Target Genes of KCMMs and HCC

**Figure 4:**
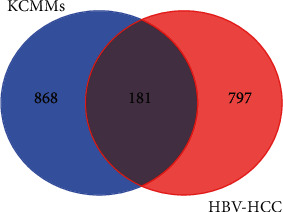
Venn chart of common target genes of KCMMs and HCC.

**Figure 5:**
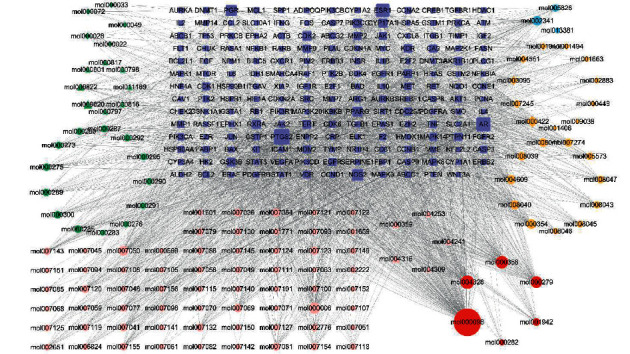
Network of KCMMs-active ingredients and common targets. Dark blue rectangles represent the target genes; orange diamonds and ellipses represent the bioactive ingredients of Zhi Zi and Yin Chen, respectively; green diamonds, triangles, and ellipses stand for the bioactive ingredients of Bai Zhu, Zhu Ling, and Fu Ling, respectively; pink ellipses and triangles stand for bioactive ingredients of Dan Shen and Yu Jin, respectively; light blue and red ellipses represent the active ingredients of Zhi Qiao and multiple drugs, respectively.

By using the Venn online tool, a total of 181 common target genes of KCMMs and HCC were identified (Figure 4). After that, the interaction network of bioactive ingredients-common target genes of KCMMs and HCC was constructed, involving 307 nodes (181 targets and 126 bioactive) and 2118 edges (interactions) (Figure 5). This network illustrates that quercetin (mol000098), naringenin (mol004328), beta-sitosterol (mol000358), cerevisterol (mol000279), kaempferol (mol000422), luteolin (mol000006), XH-14 (mol007050), isorhamnetin (mol000354), hesperetin (mol002341), eupalitin (mol008040), polyporusterone E (mol000820), etc., connect with most of the targets, which may be the important bioactive ingredients of KCMMs in the network.

### 3.6. Results of GO Term Enrichment and KEGG Pathway Analyses

**Figure 6:**
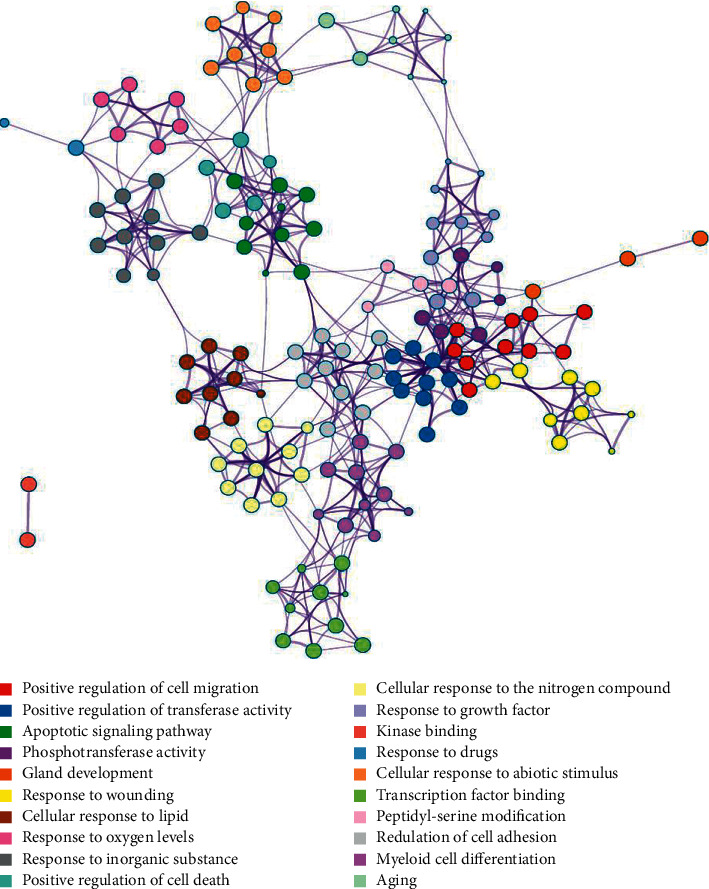
Results of the GO enrichment analysis. Each cluster is represented by a different color.

**Figure 7:**
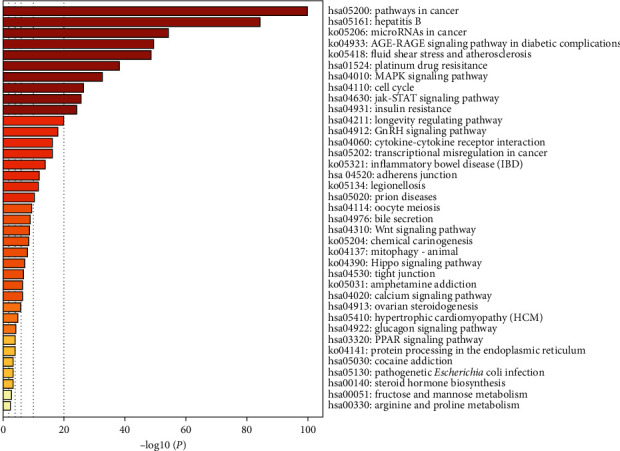
Results of KEGG pathway enrichment (*P* value <0.01).

The results of GO and KEGG analyses are demonstrated in Figures 6 and 7, respectively. Correlations between the top twenty cluster enrichment terms of GO analysis were displayed as a network (Figure 6). In Metascape, kappa scores are used as the similarity metric when performing hierarchical clustering on the enriched terms, subtrees with a similarity of >0.3 are considered a cluster, and the most statistically significant term within a cluster is chosen to represent the cluster (17). These GO enrichment terms included positive regulation of cell migration and positive regulation of transferase activity. The results of all signaling pathways of KCMMs for treating HBV-related HCC are listed in Figure 7. According to the results, the common target genes were mainly distributed in many signaling pathways such as pathways in cancer, hepatitis B, microRNAs in cancer, MAPK, JAK-STAT, Wnt, and Hippo signaling pathways, suggesting that KCMMs may treat HCC by regulating the tumor microenvironment, tumorigenesis, progression, angiogenesis, metastasis, and so on.

### 3.7. Protein-Protein Interaction Networks and Prognostic Values of Hub Genes

**Figure 8:**
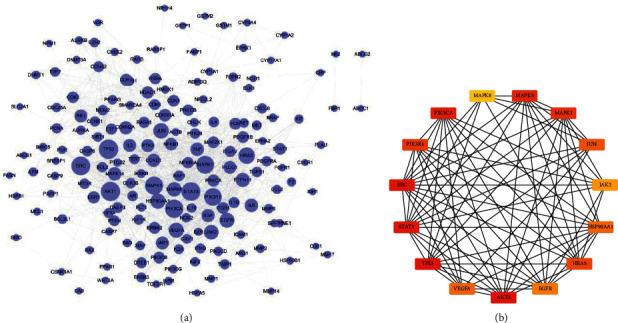
The PPI network for common target genes of KCMMs and HCC. (a) STRING PPI network (181 nodes and 1294 edges). The size of the node corresponds to the degree (number of connections) of the node. (b) Top 15 hub target genes in the PPI network based on CytoHubba of Cytoscape. The darker (red) the color, the higher the degree.

The PPI analysis of common target genes showed that the interaction network had 181 nodes and 1294 edges (Figure 8(a)). On the basis of the PPI data, the top 15 hub genes with the highest degree were identified, and the result was presented in an interaction network (Figure 8(b)). As displayed in Figure 8(b), TP53, AKT1, SRC, STAT3, MAPK3, PIK3CA, MAPK1, PIK3R1, HRAS, JUN, VEGFA, HSP90AA1, EGFR, JAK2, MAPK8, and JUN could be the potential target genes for KCMMs in the treatment of HCC. Survival analysis of hub genes in TCGA indicated that the expressions of TP53, SRC, STAT3, MAPK3, PIK3R1, HRAS, VEGFA, HSP90AA1, EGFR, and JAK2 were correlated with the prognosis of HCC (Table 4).

## 4. Discussion

In our study, the correlations between TCM syndrome types and prognosis in a retrospective cohort of HBV-related HCC patients were assessed, and our findings revealed that TCM syndrome, tumor size and number, surgery, and Child–Pugh classification were remarkably associated with the prognosis of HBV-related HCC patients, and patients with TCM excess syndrome had better OS. To further study the potential mechanisms, the network pharmacology analysis of KCMMs for treating HBV-related HCC patients with TCM excess syndrome was performed.

In HCC patients, it was reported that the TCM user group of liver cancer was remarkably associated with a reduced risk of death compared with the non-TCM user group [21, 22]. However, there is currently no prognostic analysis for different TCM syndromes of HBV-related HCC patients. In TCM, TCM syndrome (“Zheng” in Chinese) is a core guideline, and patients with the same disorders could be treated differently based on TCM syndromes [23]. In the theory of TCM, TCM syndrome is a pathological state with the information of clinical symptoms, etiologies, and disease location and characteristics, which is the basis of TCM treatment. Due to diverse and complex clinical manifestations, different patients or different disease stages may have different TCM syndromes [24]. In this study, the multivariate analysis confirmed TCM syndrome as an independent prognostic factor, and the KM analysis indicated that HBV-related HCC patients with TCM excess syndrome had the longest median survival time. Previous studies indicated that cancer patients with TCM deficiency syndrome have lower immune function and shorter survival time, while cancer patients with excess syndrome have better immune function and longer survival time, which supports the finding of our study [9]. Also, TCM can promote the antitumor effect by restoring immunosurveillance, including upregulating immunostimulatory factors and downregulating immunosuppressive factors [25]. Besides, compared with conventional therapies of cancer-associated symptoms, the incidence of related adverse events was not increased in the TCM groups [26], and a prospective cohort study showed that TCM prescriptions were safe and tolerable to most cancer patients [27]. Thus, in order to improve the prognosis of HBV-related HCC patients, Chinese materia medicas could be used to change the TCM syndrome types of HBV-related HCC patients.

Based on the data mining result of prescriptions, eight KCMMs were identified for the treatment of HCC with TCM excess syndrome. In TCM theory, it is commonly accepted that “dampness-heat,” “qi stagnation,” and “blood stasis” are the important pathogenesis of HCC. Furthermore, these eight KCMMs could play the roles of “clearing heat and promoting diuresis,” “invigorating spleen for eliminating dampness,” and “regulating qi-flowing and removing blood stasis,” which were in line with the basic theories of TCM. With the help of network pharmacology, the “drug-gene-targeted disease subtype” network can be established to clarify TCM theory [28]. In the present study, various valuable and novel ingredients and corresponding targets were found in public databases, which may have the potential to be developed for the treatment of HBV-related HCC. Additionally, a network of KCMMs-active ingredients and common targets showed a variety of key ingredients that could play a therapeutic role. A preceding study suggested that quercetin could inhibit HCC progression, which was related to the JAK2/STAT3 pathway [29]. Kaempferol and luteolin can increase the activation of caspase-3 and induce apoptosis. Also, kaempferol and luteolin are nontoxic to normal hepatocytes and are complementary antiliver cancer drugs [30]. Hesperetin nanoparticles exhibited anticancer activity by suppressing cell inflammation and proliferation in hepatocellular carcinogenesis [31]. Naringenin could inhibit vascular endothelial growth factors and regulate MAPK pathways to exert its antihepatoma effect [32]. It was reported that XH-14 could inhibit the production of a variety of inflammatory mediators and hold great promise in the treatment of inflammation-related diseases [33]. Besides, isorhamnetin may prevent liver fibrosis by inhibiting the TGF-*β*/Smad pathway and relieving oxidative stress [34]. Liver inflammation and fibrosis are considered to be the leading causes of HCC. In addition, previous studies also reported that beta-sitosterol, polyporusterone E, and eupalitin have antitumor activity [35–37]. Thus, the ingredients in KCMMs of excess syndrome are complex and diverse, but they possess strong antihepatocarcinoma activity.

In GO analysis, common target genes of KCMMs and HBV-related HCC were significantly involved in the positive regulation of cell migration, positive regulation of transferase activity, apoptotic signaling pathway, positive regulation of cell death, aging, regulation of cell adhesion, etc., which were associated with liver cancer occurrence and development. This revealed that HBV-related HCC involves abnormalities in a wide array of biological processes, and KCMMs could be used in treating HBV-related HCC by interfering with these biological processes. Furthermore, our data revealed that KCMMs may play a role in the treatment of HBV-HCC through multiple pathways which usually intersect with each other. Currently, it is considered that HBV genomes can integrate into the host genome to cause carcinogenesis, and chronic hepatitis B-associated inflammation can lead to the accumulation of genetic and epigenetic defects that are related to hepatocarcinogenesis [38]. Abnormalities of microRNAs are involved in different stages of hepatocarcinogenesis. In HBV-related HCC, microRNAs (miR-150, miR-342-3p, miR-663, miR-20b, etc.) may play a role at the transcriptional level by targeting cellular transcription factors needed for hepatocellular carcinogenesis or directly binding to HBV transcripts to affect the gene expression of HBV [39]. Yang et al. [40] used HBV probe-based capturing and next-generation sequencing to analyze HBV integration in patients with HBV and liver cancer, and the results showed that HBV integration target genes were remarkably involved in the MAPK pathway. In HBV-related HCC, Wnt-3 could induce the activation of the Wnt/*β*-catenin pathway in HCC cells, which may play a role in the carcinogenesis of HCC [41]. Infiltration of inflammatory cells in the liver caused by HBV infection is a common feature of HBV-related HCC, and these cells can secrete cytokines that act directly on liver cells. Importantly, IL-6 can activate the JAK/STAT pathway, which plays a crucial role in the occurrence and development of HCC [42]. In the Hippo signaling pathway, yes-associated protein (YAP) is the downstream effector and important oncogene, and it has been reported that YAP is involved in the hepatocarcinogenesis induced by hepatitis B virus X protein (HBx) [43]. Overall, our findings are in line with previous reports in the literature. The network based on the PPI analysis showed that 15 hub genes were identified as the most important target genes. Among them, the expressions of 10 genes, including TP53, SRC, STAT3, MAPK3, PIK3R1, HRAS, VEGFA, HSP90AA1, EGFR, and JAK2, were significantly related to the OS time of patients with HCC, which implies that KCMMs may exert an anti-HCC effect mainly through these target genes, thus prolonging the survival time of the patients. Previous research indicated that TP53 mutation could be defined as a carcinogenic driver of HBV-related HCC due to its high mutation frequency in HBV-related HCC [44]. HBV core protein can promote the development of HCC by increasing the expression of SRC and then activates the SRC/PI3K/Akt pathway [45]. Constitutive activation of STAT3 is usually observed and is closely related to tumor cell proliferation, invasion, metastasis, and angiogenesis [46]. The expression of the small protein of HBV surface antigen (HBsAg) has recently been reported to activate the JAK2/STAT3 pathway and induce the process of epithelial-mesenchymal transformation of HCC [47]. Also, increased expression of VEGFA may activate the Akt/mTOR pathway, thus promoting the occurrence of HBV-related HCC through VEGFR2 [48]. HBx could promote the invasive ability of the human HCC cell line by upregulating the expression of HSP90A [49]. Moreover, variants of EGFR genes could play a crucial role in the regulation of p21 expression and affect the prognosis of HBV-related HCC through a TP53-independent manner [50]. The expression of PIK3R1 in HCC tissues was higher than that in adjacent normal tissues, and knockdown of PIK3R1 could suppress the proliferation and migration of HCC cells [51]. Besides, HRAS gene was associated with the early invasion and metastasis of HCC caused by epithelial-mesenchymal transition [52]. Therefore, previous studies made our results more plausible. Last but not least, there are few reports about some ingredients and target genes in this paper, which may offer clues to further study potential target genes and ingredients for the treatment of HBV-related HCC. However, this paper has some potential weaknesses. In the HBV-related HCC cohort, the sample of the deficiency pattern subgroup was relatively small, which may affect the robustness of our findings. Some of the clinical data in the hospital recording system were incomplete, and the relationship between stages and prognosis was not routinely evaluated. Additionally, the mechanisms were analyzed by the network pharmacology method, but experimental verifications were deficient. Our findings should be further validated by future experiments both in vivo and in vitro.

## 5. Conclusions

Overall, associations between TCM syndromes and survival of HBV-related HCC patients were found, and patients with excess syndrome had the longest median survival time. Our results showed that KCMMs for HBV-related HCC patients with excess syndrome could play a therapeutic role by regulating targets and pathways related to the tumor microenvironment, tumorigenesis, progression, angiogenesis, invasion, metastasis, and prognosis. Thus, we speculate that expressions of oncogenes were significantly decreased, while cancer suppressor genes were increased in excess syndrome, compared with those in other syndrome types.

## Figures and Tables

**Table 1:** Results of univariate and multivariate analyses.

Characteristics	*N* (%)	Univariate analysis		Multivariate analysis	
		HR (95% CI)	*P*	HR (95% CI)	*P*
Gender					
Female	26 (12.56)	Reference	—	Reference	—
Male	181 (87.44)	0.64 (0.41–1.01)	0.06	0.55 (0.34–0.90)	0.02

Age					
<60	133 (64.25)	Reference	—	Reference	—
≥60	74 (35.75)	1.46 (1.05–2.02)	0.024	1.04 (0.73–1.49)	0.81

TCM syndrome					
Deficiency	36 (17.39)	Reference	—	Reference	—
Excess	98 (47.34)	0.39 (0.25–0.60)	<0.001	0.58 (0.36–0.95)	0.03
Intermingled	73 (35.27)	0.71 (0.46–1.10)	0.13	1.05 (0.64–1.74)	0.84

Tumor size					
<5	115 (55.56)	Reference	—	Reference	—
≥5	92 (44.44)	4.12 (2.94–5.77)	<0.001	3.99 (2.74–5.81)	<0.001

Tumor number					
Single	113 (54.59)	Reference	—	Reference	—
≥2	94 (45.41)	3.02 (2.17–4.21)	<0.001	2.13 (1.48–3.06)	<0.001

Surgery					
Yes	33 (15.94)	Reference	—	Reference	—
No	174 (84.06)	5.16 (2.63–10.15)	<0.001	4.25 (2.03–8.88)	<0.001

Child–Pugh					
A	136 (65.70)	Reference	—	Reference	—
B	52 (25.12)	2.44 (1.69–3.51)	<0.001	2.01 (0.68–1.69)	<0.01
C	19 (9.18)	4.61 (2.76–7.72)	<0.001	2.42 (0.48–2.08)	0.02

ALBI					
≤−2.6	81 (39.13)	Reference	—	Reference	—
>−2.6, ≤−1.39	103 (49.76)	2.16 (1.50–3.11)	<0.001	1.08 (0.68–1.69)	0.75
>−1.39	23 (11.11)	4.42 (2.61–7.48)	<0.001	1.00 (0.48–2.08)	0.99

HBV-DNA					
<500	76 (36.71)	Reference	—	Reference	—
≥500	131 (63.29)	2.01 (1.41–2.87)	<0.001	1.30 (0.89–1.89)	0.17

**Table 2:** Top 15 combinations of Chinese materia medicas (association rules).

The combinations of Chinese materia medicas (in Chinese, pinyin)	Num	Support %
*Atractylodes macrocephala* Koidz. (Bai Zhu), *Poria cocos* (Schw.) Wolf. (Fu Ling)	68	69.39
*Poria cocos* (Schw.) Wolf. (Fu Ling), *Atractylodes macrocephala* Koidz. (Bai Zhu)	67	68.37
*Atractylodes macrocephala* Koidz. (Bai Zhu), *Polyporus umbellatus* (Pers) Fr. (Zhu Ling)	54	55.10
*Poria cocos* (Schw.) Wolf. (Fu Ling), *Polyporus umbellatus* (Pers) Fr. (Zhu Ling)	54	55.10
*Atractylodes macrocephala* Koidz. (Bai Zhu), *Radix salviae* (Dan Shen)	51	52.04
*Poria cocos* (Schw.) Wolf. (Fu Ling), *Radix salviae* (Dan Shen)	51	52.04
*Atractylodes macrocephala* Koidz. (Bai Zhu), *Polyporus umbellatus* (Pers) Fr. (Zhu Ling), *Poria cocos* (Schw.) Wolf. (Fu Ling)	47	47.96
*Aurantii fructus* (Zhi Qiao), *Curcumae radix* (Yu Jin)	45	45.92
*Polyporus umbellatus* (Pers) Fr. (Zhu Ling), *Curcumae radix* (Yu Jin)	45	45.92
*Atractylodes macrocephala* Koidz. (Bai Zhu), *Curcumae radix* (Yu Jin)	45	45.92
*Poria cocos* (Schw.) Wolf. (Fu Ling), *Curcumae radix* (Yu Jin)	45	45.92
*Poria cocos* (Schw.) Wolf. (Fu Ling), *Polyporus umbellatus* (Pers) Fr. (Zhu Ling), *Atractylodes macrocephala* Koidz. (Bai Zhu)	44	44.90
*Atractylodes macrocephala* Koidz. (Bai Zhu), *Radix salviae* (Dan Shen), *Poria cocos* (Schw.) Wolf. (Fu Ling)	43	43.88
*Poria cocos* (Schw.) Wolf. (Fu Ling), *Radix salviae* (Dan Shen), *Atractylodes macrocephala* Koidz. (Bai Zhu)	41	41.84
*Artemisiae scopariae* Herba (Yin Chen), *Gardeniae fructus* (Zhi Zi)	40	40.82

**Table 3:** Top 30 bioactive ingredients of KCMMs according to the number of targets.

Mol ID	Ingredient (KCMMs, in Chinese pinyin)	OB	DL	TCMSP	SWISS	ETCM	SymMap	Total
Mol000098	Quercetin (Yin Chen, Zhi Zi)	46.43	0.28	146	103	57	150	271
Mol000422	Kaempferol (Zhi Zi)	41.88	0.24	57	103	72	51	198
Mol000354	Isorhamnetin (Yin Chen)	49.60	0.31	32	103	53	20	168
Mol002341	Hesperetin (Zhi Qiao)	70.31	0.27	6	104	54	5	153
Mol000275	Trametenolic acid (Fu Ling)	38.71	0.80	1	89	80	1	152
Mol008040	Eupalitin (Yin Chen)	46.11	0.33	12	100	51	10	150
Mol004328	Naringenin (Yu Jin, Zhi Qiao)	59.29	0.21	35	92	44	35	150
Mol004609	Areapillin (Yin Chen)	48.96	0.41	13	100	48	—	148
Mol008039	Isoarcapillin (Yin Chen)	57.40	0.41	11	101	42	7	145
Mol000006	Luteolin (Dan Shen)	36.16	0.25	54	23	72	55	143
Mol000300	Dehydroeburicoic acid (Fu Ling)	44.17	0.83	—	67	79	—	133
Mol003095	Corymbosin (Zhi Zi)	51.96	0.41	21	103	—	13	122
Mol008041	Eupatolitin (Yin Chen)	42.55	0.37	8	73	44	7	120
Mol007155	Tanshinone IIb (Dan Shen)	65.26	0.45	12	105	—	10	118
Mol007120	Miltionone II (Dan Shen)	71.03	0.44	7	109	1	6	117
Mol007081	Danshenol B (Dan Shen)	57.95	0.56	6	110	5	6	116
Mol004561	Sudan III (Zhi Zi)	84.07	0.59	11	106	0	7	113
Mol007069	Przewaquinone C (Dan Shen)	55.74	0.40	20	98	0	11	112
Mol007077	Sclareol (Dan Shen)	43.67	0.21	1	111	0	1	112
Mol004316	1,7-Diphenyl-3-acetoxy-6(E)-hepten (Yu Jin)	48.47	0.22	—	111	—-	—	111
Mol000279	Cerevisterol (Fu Ling, Zhu Ling)	37.96	0.77	1	108	0	1	110
Mol007274	Skrofulein (Yin Chen)	30.35	0.30	8	103	0	7	110
Mol007041	2-Isopropyl-8-methylphenanthrene-3,4-dione (Dan Shen)	40.86	0.23	31	79	13	17	109
Mol007245	3-Methylkempferol (Zhi Zi)	60.16	0.26	9	103	0	6	109
Mol007050	XH-14 (Dan Shen)	62.78	0.40	10	106	0	7	106
Mol000358	Beta-sitosterol (Yu Jin, Zhi Qiao, Zhi Zi, Yin Chen)	36.91	0.75	35	41	44	14	106
Mol000822	Polyporusterone G (Zhu Ling)	33.43	0.81	1	106	0	1	106
Mol007036	Arucadiol (Dan Shen)	33.77	0.29	15	89	0	13	105
Mol008047	Artepillin A (Yin Chen)	68.32	0.24	16	92	0	12	104
Mol000285	Polyporenic acid C (Fu Ling)	38.26	0.82	—	103	—	—	103

DL: drug-likeness; OB: oral bioavailability; SWISS: SwissTargetPrediction. Mol IDs are from TCMSP.

**Table 4:** Prognostic values of top 15 hub target genes (common targets of KCMMs and HCC).

No.	Gene name	Low	High	HR (95%)	*p* value
1	TP53	90	274	0.62 (0.45–0.96)	0.029^*∗*^
2	AKT1	272	92	1.42 (0.99–2.04)	0.057
3	SRC	258	106	1.75 (1.22–2.53)	0.0023^*∗*^
4	STAT3	264	100	0.56 (0.36–0.87)	0.0093^*∗*^
5	PIK3CA	272	92	1.33 (0.91–1.94)	0.13
6	MAPK3	248	116	1.9 (1.34–2.69)	0.00025^*∗*^
7	MAPK1	98	266	1.19 (0.81–1.77)	0.37
8	PIK3R1	201	163	0.47 (0.32–0.68)	3.7*e* − 05^*∗*^
9	HRAS	208	156	1.51 (1.07–2.13)	0.019^*∗*^
10	JUN	181	183	1.18 (0.83–1.66)	0.36
11	HSP90AA1	137	227	1.77 (1.21–2.6)	0.0028^*∗*^
12	VEGFA	268	96	1.74 (1.21–2.5)	0.0025^*∗*^
13	EGFR	91	273	0.61 (0.43–0.89)	0.0085^*∗*^
14	JAK2	126	238	0.67 (0.47–0.95)	0.023^*∗*^
15	MAPK8	108	256	0.76 (0.53–1.1)	0.14

HR = hazard rate; ^*∗*^*p* value <0.05.

## Data Availability

The data obtained in this research can be made available from the corresponding author upon request or from the indicated sources.

## Supplementary Materials

Supplementary Materials Supplementary Table S1: distribution of TCM syndromes. Supplementary Table S2: the frequency of 124 Chinese materia medicas (in Chinese name). Supplementary Table S3: the detailed data of bioactive ingredients of KCMMs and target genes.
