# Supplementary material for: Determining the Traditional Chinese Medicine (TCM) Syndrome with the Best Prognosis of HBV-Related HCC and Exploring the Related Mechanism Using Network Pharmacology
Source: Evid Based Complement Alternat Med. 2021 Jun 29;2021:9991533. doi: 10.1155/2021/9991533 (PMC8263254; doi:10.1155/2021/9991533)
Supplement: Supplementary Materials — Supplementary Table S1: distribution of TCM syndromes. Supplementary Table S2: the frequency of 124 Chinese materia medicas (in Chinese name). Supplementary Table S3: the detailed data of bioactive ingredients of KCMMs and target genes. [file 9991533.f1.zip › 9991533.f1/Supplementary Table S1(new).pdf]

Characteristics	Deficiency		Excess		Intermingled	
	N	%	N	%	N	%
Gender (Female)	6	16.67%	8	8.16%	12	16.44%
Male	30	83.33%	90	91.84%	61	83.56%
Age (<60)	27	75.00%	62	63.27%	44	60.27%
>=60	9	25.00%	36	36.73%	29	39.73%
Tumor size (<5)	11	30.56%	67	68.37%	37	50.68%
>=5	25	69.44%	31	31.63%	36	49.32%
Tumor number (single)	17	47.22%	58	59.18%	38	52.05%
>=2	19	52.78%	40	40.82%	35	47.95%
Surgery (Yes)	5	13.89%	15	15.31%	13	17.81%
No	31	86.11%	83	84.69%	60	82.19%
Child-Pugh (A)	15	41.67%	71	72.45%	50	68.49%
B	13	36.11%	24	24.49%	15	20.55%
C	8	22.22%	3	3.06%	8	10.96%
ALBI (⩽-2.6)	10	27.78%	47	47.96%	24	32.88%
>-2.6, <=-1.39	17	47.22%	44	44.90%	42	57.53%
>-1.39	9	25.00%	7	7.14%	7	9.59%
HBV DNA (<500)	11	30.56%	39	39.80%	26	35.62%
>=500	25	69.44%	59	60.20%	47	64.38%
